# Physical activity and sedentary behavior during the early years in Canada: a cross-sectional study

**DOI:** 10.1186/1479-5868-10-54

**Published:** 2013-05-04

**Authors:** Rachel C Colley, Didier Garriguet, Kristi B Adamo, Valerie Carson, Ian Janssen, Brian W Timmons, Mark S Tremblay

**Affiliations:** 1 Health Analysis Division, Statistics Canada, Ottawa, Canada; 2 Healthy Active Living and Obesity Research Group, Children’s Hospital of Eastern Ontario Research Institute, Ottawa, Canada; 3 Faculty of Physical Education and Recreation, University of Alberta, Edmonton, Canada; 4 School of Kinesiology and Health Studies, Queen’s University, Kingston, Canada; 5 Child Health & Exercise Medicine Program, McMaster University, Hamilton, Canada

**Keywords:** Preschool, Pediatric, Surveillance, Ambulation, Step counts

## Abstract

**Background:**

Physical activity and sedentary behavior habits are established during early childhood, yet only recently has objectively measured data been available on children aged 5 years and younger. This study presents data on the physical activity and sedentary behaviors of Canadian children aged 3–5 years.

**Methods:**

Data were collected as part of the Canadian Health Measures Survey between 2009 and 2011. A nationally-representative sample (n = 459) of children aged 3–5 years wore Actical accelerometers during their waking hours for 7 consecutive days. Data were collected in 60-sec epochs and respondents with ≥4 valid days were retained for analysis. Parents reported their child’s physical activity and screen time habits in a questionnaire.

**Results:**

Eighty-four percent of 3–4 year old children met the physical activity guideline of 180 minutes of total physical activity every day while 18% met the screen time target of <1 hour per day. Fourteen percent of 5 year old children met the physical activity guideline of 60 minutes of daily moderate-to-vigorous physical activity (MVPA) while 81% met the screen time target of <2 hours per day. Children aged 3–4 years accumulated an average of 352 min/d of total physical activity and 66 minutes of MVPA while 5 year old children accumulated an average of 342 min/d of total physical activity and 68 minutes of MVPA. Children were sedentary for approximately half of their waking hours and spent an average of 2 hours per day in front of screens. Only 15% of 3–4 year olds and 5% of 5 year olds are meeting both the physical activity and sedentary behavior guidelines.

**Conclusions:**

Promoting physical activity while reducing sedentary behavior is important at all stages of life. The findings of the present study indicate that there remains significant room for improvement in these behaviors among young Canadian children.

## Background

The early years represent a critical period for the establishment of active living habits; however, little is known about how much physical activity and sedentary behavior young children are accumulating. Research to date suggests that children less than 5 years of age spend a small proportion of time being active and have high levels of inactivity [1-3], although most studies historically focused on moderate-to-vigorous-intensity physical activity (MVPA) and relied on non-objective measures. A recent systematic review reported that physical activity during the early years is associated with improved measures of adiposity, motor skill development, psychosocial health, and cardiometabolic health indicators [4]. High levels of sedentary behavior in this age group, in particular high levels of television viewing, is associated with increased adiposity and lower measures of psychosocial and cognitive development [5].

The National Association for Sport and Physical Education (NASPE) recommends at least one hour of structured and one or more hours of unstructured physical activity every day for children from birth to age 5 years [6]. Levels of adherence to the NASPE guidelines have varied considerably (32-79%) and this is likely due in part to differences in measurement tools and inconsistent inclusion of light intensity physical activity [1,3,7]. Australian and Canadian guidelines recommend that young children (aged 1–4 years) participate in physical activity of any intensity (i.e., light, moderate or vigorous) for at least 3 hours a day [8,9]. The Canadian guidelines also recommend progression toward at least 60 minutes of energetic play (i.e., MVPA) by 5 years of age to align with the guidelines for children ages 5 to 17 years which recommend 60 minutes of MVPA per day [9]. Compliance with the recommendation of at least 180 min of physical activity at any intensity also varies considerably between countries, ranging from 5% in a sample of children from Melbourne, Australia [1] to 73% in a sample of children from Hamilton, Canada [10].

The American Academy of Pediatrics (AAP) recommends no more than 2 hours per day of television time for children aged 2 years and older [11] and the Australian Government’s Department of Health and Ageing stipulates no more than one hour of screen-based entertainment per day for 3 to 5 year old children [8]. In a sample of 3 to 5 year old children from Melbourne, Australia, 22% met the Australian recommendation (<1 hr/d) and 59% met the AAP recommendation (<2 hr/d) [1]. New Canadian sedentary behavior guidelines were released in 2012 [12]. Key aspects of the Canadian sedentary behavior guidelines are that i) children under age 2 do not engage in screen time, a recommendation consistent with the American Association of Pediatrics [11], and ii) screen time be limited to less than 1 h per day in 3 to 4 year old children [12], and less than 2 h per day in 5 year old children [13]. Data from a sample of children from Kingston, Canada indicated that less than half (46%) of children aged 2–4 years met the Canadian screen time recommendation of <1 hr/d [14]. It is presently unknown whether this finding is representative of children in this age group across Canada.

The measurement of physical activity and sedentary behavior in children during the early years is needed not only to estimate the proportion of the population meeting recommendations pertaining to physical activity and sedentary behavior guidelines, but to establish the relationships between these movement constructs with health outcomes, and to enable researchers to evaluate the effectiveness of interventions. Physical activity and sedentary behavior in young children can be measured using indirect (e.g., parent-report, direct observation) and direct (e.g., pedometers and accelerometers) methods. Accelerometers provide objective information on the frequency, intensity and duration of movement. Parent-reported information provides important contextual information on the specific behaviors young children are engaging in while being active or sedentary [15].

It is presently unknown what proportion of a nationally representative sample of Canadian children aged 3 to 5 years is meeting these new physical activity and sedentary behavior guidelines. The 2^nd^ cycle of the Canadian Health Measures Survey (CHMS) collected accelerometry and parent-reported data on physical activity and sedentary behavior on a sample of children aged 3–5 years. The purpose of this paper is to report, for the first time ever on a nationally-representative sample, the physical activity and sedentary behaviors of Canadian children aged 3–5 years.

## Methods

The CHMS collected data from a nationally representative sample of the population aged 3 to 79 years living in private households at the time of the survey [16]. Residents of Indian Reserves, institutions and certain remote regions, and full-time members of the Canadian Armed Forces were excluded. Approximately 96% of Canadians were represented. The survey involved an interview in the respondent’s home and a visit to a mobile examination center for a series of physical measurements. Data were collected at 18 sites across Canada from August 2009 to November 2011. Ethics approval to conduct the CHMS was obtained from Health Canada’s Research Ethics Board [17]. For younger children, a parent or legal guardian provided written consent and written assent was also obtained from the child. Participation was voluntary; respondents could opt out of any part of the survey at any time.

Upon completion of the mobile examination center visit, ambulatory respondents were asked to wear an Actical accelerometer (Phillips – Respironics, Oregon, USA) over their right hip on an elasticized belt during their waking hours for 7 consecutive days. The Actical (dimensions: 2.8 × 2.7 × 1.0 centimetres; weight: 17 grams) measures and records time-stamped acceleration in all directions, providing an indication of movement intensity, duration and frequency. The digitized values are summed over a user-specified interval of 60-sec, resulting in a count value per minute (cpm). Accelerometer signals are also recorded as steps per minute. The Actical has been validated to measure physical activity and sedentary behavior in preschool aged children [18,19].

The monitors were initialized to start collecting data at midnight following the mobile examination center appointment. Respondents were blind to all data while they wore the device. The monitors were returned to Statistics Canada in a prepaid envelope, where the data were downloaded and the monitor was checked to determine if it was still within the manufacturer’s calibration specifications [20]. Standard data reduction procedures were followed that are consistent with cycle 1 of the CHMS [20,21]. A valid day for this age group was defined as 5 or more hours of monitor wear time [22] and respondents with 4 or more valid days were retained for analyses [20,21,23]. Wear time was determined by subtracting non-wear time from 24 hours. Non-wear time was defined as at least 60 minutes of consecutive minutes of zero counts, with allowance for 1 to 2 minutes of counts between 0 and 100 [22,23].

For this study, time spent at various levels of movement intensity (sedentary, light, moderate, vigorous) was based on cut-points corresponding to each intensity level. The cut-point used for MVPA was 1,150 cpm [18]. A cut-point of 100 cpm was used to delineate sedentary behavior from light physical activity [24]. Children aged 3–4 years were classified as meeting the guideline if they achieved 180 min of physical activity at any intensity every day (i.e., 180 minutes ≥ 100 counts per minute) on all valid days (e.g. “daily”). To determine the probability that 5 year old children accumulated at least 60 minutes of MVPA at least 6 days a week, the analytical approach was harmonized with that used previously in 6–19 year old children in the CHMS [21]; an approach that was based on the technique used in the United States to analyze the 2003 to 2004 National Health and Nutritional Examination Survey (NHANES) accelerometry data [23]. To maximize the sample size, a Bayesian approach was used to incorporate the information from children with 4 or more valid days. An individual’s probability of being active at least 6 days out of 7 days was estimated using a Beta distribution for the observed combination of active and wear days. The estimated population prevalence is the weighted average of these individual probabilities [25]. Progression towards meeting the physical activity guidelines of 60 daily minutes of MVPA on valid days in 3–4 year olds was assessed using the same Bayesian approach to examine the proportion of 3–4 year olds who accumulated 180 minutes of physical activity at any intensity where 10, 20, 30, 45 and 60 minutes of that time was at least MVPA. Average daily step counts were calculated and the proportions of children accumulating an average of 6,000 steps per day and 6,000 steps on every valid day [10] were both assessed.

As part of the CHMS household questionnaire, parents were asked a series of questions about their child’s level of physical activity and engagement in sedentary behaviors:

•Over the past 7 days, on how many days was he/she physically active for a total of at least 60 minutes per day? (none, 1 day, 2–3 days, 4 days or more)

•Over a typical or usual week, on how many days is he/she physically active for a total of at least 60 minutes per day? (none, 1 day, 2–3 days, 4 days or more)

•About how many hours a week does he/she usually take part in physical activity (that makes him/her out of breath or warmer than usual) outside of school while participating in lessons or league or team sports? (never, <2 hrs/wk, 2–3 hrs/wk, 4–6 hrs/wk, 7+ hrs/wk)

•About how many hours a week does he/she usually take part in physical activity (that makes him/her out of breath or warmer than usual) outside of school while participating in unorganized activities, either on his/her own or with friends? (never, <2 hrs/wk, 2–3 hrs/wk, 4–6 hrs/wk, 7+ hrs/wk)

•On average, about how many hours a day does he/she watch TV or videos or play video games? (doesn’t watch TV or videos or play video games, <1 hr/d, 1–2 hrs/d, 3–4 hrs/d, 5–6 hrs/d, 7+ hrs/d)

•On average, about how many hours a day does he/she spend on a computer (working, playing games, e-mailing, chatting, surfing the internet, etc.)? (doesn’t use a computer, <1 hr/d, 1–2 hrs/d, 3–4 hrs/d, 5–6 hrs/d, 7+ hrs/d)

Time spent watching TV, videos or playing video games and time spent on a computer was derived using the mid-points of the previous category (i.e. 0, 0.5, 1.5, 2.5, 5.5 and 7 hours for the respective categories). The amount of time was summed for the two questions to obtain screen time and children aged 3 to 4 with ≤1 h/d of screen time or children aged 5 with ≤2 h/d of screen time were deemed as following the screen time recommendations within the sedentary behavior guidelines. For example, if a parent reported <1hr/d for both the question about TV/videos and the question about computer time, that child was assigned the midpoint value of 0.5 hr/d for each to give a total equal to 1 hr/d of screen time, which is slightly different to <1 hr/d. This way of deriving screen time means that we assessed whether a child is accumulating ≤1 hr of screen time per day rather than the actual guideline which is <1 hr/d.

The response rate for selected household was 75.9%, meaning that in 75.9% of these households, a resident provided the sex and date of birth of all household members. One or two members of each responding household were chosen to participate in the CHMS; 92.6% of the parents of selected 3–5 year olds completed the household questionnaire, and 79.4% of this group participated in the mobile examination center component. Five children did not accept the activity monitor and 48 never returned the monitor. Of the children who participated in the mobile examination center component, 76.9% wore the accelerometer for at least 4 valid days. After adjusting for the sampling strategy, the final response rate for having a minimum of 4 valid days was 42.7% (75.9 × 92.6 × 79.4 × 76.9). This article is based on 459 examination center respondents aged 3–5 years who provided a minimum of 4 days of valid accelerometer data.

All analyses were completed using SAS version 9.2 and were based on weighted data for respondents with at least 4 valid days of data. To account for survey design effects of the CHMS, standard errors, coefficients of variation, and 95% confidence intervals were estimated using the bootstrap technique [26-28].

## Results

Characteristics of the 459 children included in the analysis are in Table 1. The average age of the sample was 4 years and the sex split was almost equal (50.5% were boys). The majority (83%) of the sample was considered healthy weight according to the International Obesity Task Force classification cut-offs [29].

**Table 1:** Selected characteristics of weighted sample, by sex, household population aged 3–5 years, Canada, August 2009 to November 2011

**Characteristics**	**All**		**Boys**		**Girls**	
	**Estimate**	**S.E.**	**Estimate**	**S.E.**	**Estimate**	**S.E.**
Total sample (number)	459		232		227	
Age (years)	4.0	0.04	4.0	0.09	4.0	0.07
Height (cm)	106.4	0.43	107.0	0.64	105.7	0.91
Weight (kg)	18.6	0.19	19.0	0.33	18.1	0.39
BMI (kg/m^2^)	16.3	0.10	16.5	0.14	16.0	0.14
BMI category						
Healthy weight(%)	83.0	2.8	84.7	3.3	81.3	3.9
Overweight/obese(%)	16.4^E^	2.9	14.3^E^	3.4	18.7^E^	3.9

E Interpret with caution.

International Obesity Task Force thresholds used to define Overweight/Obese.

### Meeting the physical activity guidelines based on accelerometer data

**Figure 1:**
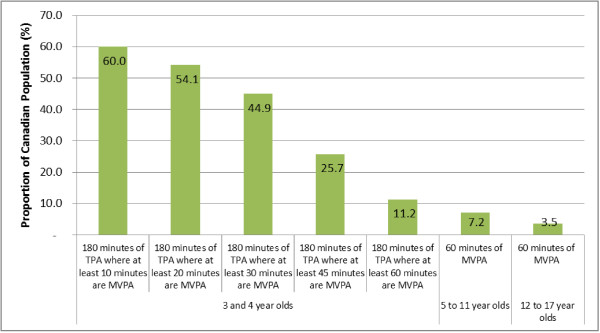
**Compliance with moderate-to-vigorous physical activity targets, household population aged 3–17 years, Canada, August 2009 to November 2011.** Note: The bars representing the 5–11 and 12–17 age ranges are included to provide context for the data presented on 3–4 year olds in this figure. The source of the data is the same for the entire age range: Cycle 2 (2009–2011) of the Canadian Health Measures Survey, Statistics Canada.

Eighty-four percent of 3 and 4 year olds met the current physical activity guideline, defined as being active at any intensity for at least 180 minutes every day (Table 2). Ninety-eight percent were active on all valid days except one. Progression towards accumulating 60 minutes of daily MVPA as part of the 180 minutes of total physical activity (3–4 year olds) is presented in Figure 1. More than half of 3 and 4 year olds accumulated at least 20 minutes of MVPA within their 180 minutes per day of total physical activity while 11% accumulated at least 60 minutes of MVPA within their 180 minutes of total physical activity (Figure 1). Fourteen percent of 5 year olds accumulated at least 60 minutes of MVPA on at least 6 days per week (the operational definition of meeting the guideline of 60 minutes of MVPA every day).

**Table 2:** Adherence to physical activity and sedentary behaviour guidelines, household population aged 3 to 5 years, Canada, August 2009 to November 2011

			**95% CI**	
**Age**	**Target**	**%**	**From**	**To**
*Physical activity*				
3 to 4 years	180 minutes of total physical activity, all valid days	83.8	77.7	90.0
	≥ 6,000 steps per day (average)	91.8	86.1	97.5
	≥ 6,000 steps per day (all valid days)	44.7	33.7	55.6
5 years	At least 60 minutes of MVPA on at least 6 days	13.7	9.4	18.0
	≥ 6,000 steps per day (average)	87.1	76.7	97.4
	≥ 6,000 steps per day (all valid days)	44.8	32.2	57.5
*Sedentary behaviour*				
3 to 4 years	≤1 hr of parent-reported average daily screen time	17.9	11.9	23.8
5 years	≤2 hr of parent-reported average daily screen time	80.7	71.2	90.2
*Both guidelines*				
3 to 4 years	≥180 minutes of total physical activity and ≤1 hr/d screen time	15.3	10.3	20.4
5 years	≥60 minutes of MVPA and ≤2 hr/d screen time	5.3	2.2	8.3

### Meeting the screen time recommendation within the sedentary behavior guidelines

Eighteen percent of children aged 3 and 4 years met the screen time recommendation within the sedentary behavior guidelines, which states that children of this age should accumulate less than 1 hour per day of screen time [12] (Table 2). Eighty-one percent of 5 year-old children met the screen time recommendations, which states that children of this age should accumulate less than 2 hours per day of screen time (Table 2). Fifteen percent of 3–4 year olds and 5% of 5 year olds met both the physical activity and the sedentary behavior guidelines.

### Average daily physical activity and sedentary behavior based on accelerometer data

On average, 3 and 4 year-old children accumulated 352 daily minutes of total physical activity and spent 50% (~5.8 hrs∙d^-1^) of their waking time per day engaged in sedentary behavior, 41% (~4.8 hrs∙d^-1^) engaged in light intensity physical activity and 9% (~66 min∙d^-1^) of their day engaged in MVPA (Table 3). On average, 5 year old children accumulated 343 daily minutes of total physical activity and spent 53% (~6.4 hrs∙d^-1^) of their waking time per day engaged in sedentary behavior, 38% (~4.6 hrs∙d^-1^) engaged in light intensity physical activity and 9% (~68 min∙d^-1^) of their day engaged in MVPA (Table 3). The average accelerometer wear time for all children in the sample was approximately 12 hours per day.

**Table 3:** Average daily minutes of activity at various levels of intensity and average daily step counts, by age and weight status, household population aged 3 to 5 years, Canada, August 2009 to November 2011

	**Sedentary time**				**Light physical activity**				**MVPA**				**Total physical activity**				**Step counts**			
	**min/d**	**S.E.**	**95% CI**		**min/d**	**S.E.**	**95% CI**		**min/d**	**S.E.**	**95% CI**		**min/d**	**S.E.**	**95% CI**		**steps/d**	**S.E.**	**95% CI**	
3-4 years	348	7	332	365	285	5	275	296	66	3	61	72	352	6	340	364	9764	348	9012	10516
5 years	381*	9	361	400	275	6	261	289	68	3	62	74	343	8	325	361	10202	578	8954	11450
Healthy weight	358	7	342	375	281	4	273	290	68	2	62	73	349	5	337	361	9941	337	9214	10669
Overweight/Obese	360	9	341	379	287	11	263	311	63	5	52	74	350	15	318	382	9753	678	8290	11217

MVPA, moderate-to-vigorous physical activity.

CI, confidence interval.

* Significantly different to 3–4 year old children.

### Daily step counts

Children aged 3 and 4 years accumulated an average of 9,764 step counts per day while 5 year old children accumulated an average of 10,202 step counts per day (Table 3). The proportion of children meeting the 6,000 steps per day target is presented in Table 2.

### Parent-reported physical activity and sedentary behavior

Parents reported that their 3 and 4 year old children are physically active for at least 60 minutes per day on an average of 5 days a week. Parents of 3 and 4 year old children reported more unorganized compared to organized physical activity (5.3 vs. 0.6 hrs∙wk^-1^). The average weekly time 3 and 4 year olds spent in front of screens was 2.2 hrs∙wk^-1^(1.9 hrs∙wk^-1^ watching TV/video and 0.3 hrs∙wk^-1^on computer). Parents reported that their 5 year old children were physically active for at least 60 minutes per day on an average of 5 days per week and engaged in more organized activity compared to younger children (1.0 vs. 0.6 hr∙wk^-1^) and similar levels of unorganized activity (5.2 vs. 5.3 hr∙wk^-1^). The average weekly time 5 year olds spent in front of screens was 1.9 hr∙wk^-1^(1.4 hrs∙wk^-1^ watching TV/video and 0.5 hrs∙wk^-1^on computer).

## Discussion

The majority of Canadian children aged 3–4 years are meeting the current physical activity guideline of at least 180 minutes of physical activity at any intensity every day [9]. This proportion is much higher when compared to older children in Canada, for whom the guideline focuses on MVPA and not total physical activity. According to the CHMS, 14% of 5 year olds met the physical activity guideline of 60 daily minutes of MVPA [30] while 7% of 5 to 11 year-olds and 3.5% of 12 to 17 year-olds met this same guideline [31]. Eighteen percent of 3–4 year olds and 81% of 5 year olds met the screen time recommendation within the current sedentary behavior guidelines, which recommend less than 1 hour a day of screen time in 2–4 year olds [12] and no more than 2 hours a day of screen time from age 5 to 17 [13]. Only 15% of 3–4 year olds and 5% of 5 year olds met both the physical activity and sedentary behavior guidelines (Table 2).

The difference in the proportion meeting the physical activity guidelines between ages 3/4 years to 5 years is largely explained by the shift in volume and intensity of physical activity recommended by the different physical activity guidelines, from 180 minutes of physical activity at any intensity to 60 minutes of daily MVPA. In accelerometer data analysis, light physical activity is defined as any observations above the sedentary cut-point (100 cpm) and therefore makes up a large proportion of the day. For example, 3 and 4 year-old children in the present analysis spent 41% (over 4 hours) of their waking hours engaged in light physical activity, and almost all children achieved the recommended 180 minutes of total physical activity every day. The physical activity guideline for children aged 0 to 4 years also states that children should progress towards accumulating 60 minutes of daily MVPA by age 5 [9]. To assess how 3 to 4 year olds were progressing towards this target, we examined the proportion that were accumulating 180 minutes of total physical activity with the added stipulation that at least 10, 20, 30, 45 or 60 minutes were of at least moderate intensity (Figure 1). The added 60 min MVPA requirement brought the 3 and 4 year olds to a level of meeting the guideline (11%) that was more consistent with children aged 5–11 years (7%). Most young children aged 3 and 4 years of age were active enough to meet the guideline; however, based on these CHMS data, the progression towards accumulating 60 minutes of daily MVPA by age 5 appears to be occurring only in a small proportion of this nationally representative sample of Canadian children.

Placing the findings of the current study in context with previous work is limited by the use of different models of accelerometers between studies, different choices of intensity cut-points, and in particular, the use of varying epoch lengths. The current study used a 60-sec epoch which is much longer than previous studies in this age group [1,10,32,33]. The encouraging physical activity result observed for 3 and 4 year children in the present analysis (84% meeting the physical activity guideline) is consistent with other data from Hamilton, Canada that reported between 73-100% of children accumulated at least 180 minutes per day of total physical activity [10,32]. This is in contrast to a recent study based in Melbourne, Australia that reported only 5% met the recommended minimum level of 3 hours per day of total physical activity [1]. The current study observed higher levels of total physical activity (343–352 min/d) compared to the two studies based in Hamilton, Ontario (252 and 220 min/d) [10,32] and the study based in Melbourne, Australia (127 min/d) [1]. However, in comparison to the Hamilton, Canada studies [10,30], children in the current study accumulated less MVPA (66–68 vs 75–92 min∙d^-1^). Further, Gabel and colleagues reported that 57% accumulated 60 minutes of MVPA within their 180 minutes of total physical activity [10] whereas in the present analysis, only 11% achieved the same. Hinkley and colleagues did not report daily minutes of MVPA but reported that MVPA made up a smaller proportion of waking hours compared to the current study (3.4 vs 9.5%) [1]. The higher levels of MVPA observed in the two Hamilton, Canada studies [10,32] are consistent with a study by Vale and colleagues which observed that 93.5% and 77.6% of children accumulated 60 min∙d^-1^ of MVPA on weekdays and weekends, respectively [33]. It has been suggested that longer epochs may underestimate MVPA and overestimate light intensity physical activity, especially in very young children [32,34]. The reason for this is the inability of longer epochs to capture the sporadic and intermittent nature of activity that is typical in this age group [3]. This may partially explain why the daily minutes of MVPA were lower in the current study compared to the two Hamilton, Canada studies; however, this does not explain why Hinkley and colleagues reported such low MVPA values as they also used an epoch of 15 sec. Additional differences in chosen intensity cut-points between studies may be contributing to differences in findings. Specifically, the cut-point used to delineate sedentary from light intensity physical activity in the study from Melbourne, Australia [1] was higher than other studies using the same monitor, thus leading to lower light and total physical activity. These inconsistencies in methodological design highlight the need for data harmonization to fully understand physical activity prevalence during the early years across countries.

The average daily step counts observed in the present analysis (3–4 year olds: 9,764; 5 year olds: 10,202 steps per day) are consistent with other step count data for this age group collected with both accelerometers (8,968 steps per day) [10] and pedometers (9,980 steps per day) [35]. Recently, a daily step target of 6,000 was proposed for the early years to be used as a step target equating to 180 minutes of total physical activity where 60 minutes are MVPA [10]. In the present study, the majority of children accumulated a weekly average of at least 6,000 steps per day (3–4 year olds: 92%, 5 year olds: 87%) while fewer achieved this target on all valid days (45%). This finding highlights the limitation of a weekly average step count value to identify children who are meeting a daily target. In the current study, 84% of 3–4 year olds met the physical activity guideline according to the accelerometer results. If only step count data had been available, we would have concluded that 45% were meeting the daily guideline. Further research is needed to examine the relationships between accelerometer- and pedometer-measured physical activity at very young ages when gait patterns are still being established.

In the present analysis, only 18% of children aged 3–4 years met the sedentary behavior guideline of less than 1 h per day of screen time. Carson and colleagues reported a higher proportion (43%) of children meeting the same sedentary behavior guideline in 2–4 year old children from the Kingston, Canada health region [14]. The questions asked of parents to derive screen time were very similar between these two studies. Participants in the Kingston, Canada study were recruited primarily from registered child care centers while the CHMS recruited from a broader population that included those registered and not registered in these types of programs. It is possible that the Kingston, Canada sample reflected children of a higher socioeconomic status and with more structured days who had fewer opportunities to accumulate screen time. Also, the CHMS reflects the entire Canadian population instead of a single health region. Other countries have assessed a similar screen time target and found results consistent with ours. For example, 22% of 3 to 5 year old Australian children accumulated 1 h or less of daily screen time [1]. At present the sedentary behavior guideline do not stipulate a total daily sedentary time target (i.e., that encompasses sedentary activities beyond screen time). A lower proportion of the waking hours was spent in sedentary time in the present population of 3 to 5 year old children (50%) when compared to older children (63%) [31] and adults (71%) [31] in the CHMS, indicating that this age group is the least sedentary age group in the Canadian population.

Determining the proportion of children meeting physical activity guidelines using accelerometry data is largely dependent on the accelerometer cut-points used to define different levels of intensity. The MVPA cut-point recently proposed by Adolph and colleagues [18] was used in the present analysis because it was developed for Actical accelerometer data collected in 60-sec epochs in 3–5 year old children. Further, this cut-point (1,150 cpm) was based on an activity energy expenditure value of 0.05 kcal∙kg^-1^∙min^-1^ or approximately 2–3 metabolic equivalents (METS); a demarcation point consistent with that used for the moderate cut-point (≥0.04 kcal∙kg^-1^∙min^-1^; 1,500 cpm) used in older children and youth in the CHMS [21,36]. The only other published Actical cut-point for this age group was designed for use with 15-sec epoch data and was based on a very different methodological approach that placed the demarcation point between light and moderate at an energy expenditure level of 20 ml∙kg^-1^∙min^-1^ or approximately 5.7 METS [19]. While the appropriateness of using METS to define energy expenditure in young children has been questioned [19,37], the large gap in MET values would have rendered the physical activity estimates from 3 to 5 year old children not comparable to older children in the CHMS. Had we used the cut-point presented by Pfeiffer and colleagues (multiplied by 4 to work with 60-sec epoch data: 2,860 cpm), the physical activity estimates would have been markedly different. For example, instead of a daily average of 68 minutes in 5 year old children, we would have reported only 17 minutes of daily MVPA. Further, this would have resulted in only 0.5% of 5 year olds meeting the physical activity guideline compared to the 14% reported herein. These examples highlight the considerable impact cut-point values can have on physical activity estimates.

The results presented here provide the first estimates of the proportion meeting the physical activity and sedentary behavior guidelines on a nationally representative sample of Canadian children aged 3–5 years. The accelerometry data provide objective estimates and the parent-reported data provide important information about screen time behaviors and the context within which physical activity is accumulated in this age group (e.g., the breakdown between organized and unorganized activity). Consistent with the accelerometer data, parents reported high levels of physical activity participation in their children. As expected, time spent in unorganized activities (i.e., unstructured free play) was higher than organized activities in the younger children. Parent-reported data may be impacted by recall and social desirability bias [38] and the questions used in this survey have not undergone rigorous validation testing. Accelerometers are limited in their ability to capture some activities (e.g., swimming, cycling, load bearing, incline changes) which may lead to some underestimation of overall activity. Further, the 60-sec epoch used for data collection in the CHMS may be an additional cause of underestimation in levels of MVPA and overestimation of light intensity physical activity [32,34]. Seasonal variation could not be assessed in this sample; however, this issue is of relevance in the Canadian context [39] and should be explored in larger data sets. An important area of future research would be to examine whether enrolment in structured childcare programs impacts upon physical activity and sedentary behavior in very young children. This could not be assessed within this study because a specific question relating to childcare arrangement was not asked as part of the household survey. Also of interest would be to examine differences in physical activity and sedentary behavior between healthy weight and overweight children. This was not possible in the present analysis because of the sample size.

The transition in guidelines between age 4 and 5 years creates two challenges: i) interpreting the transition from 84% meeting the guideline at age 3–4 years to 14% at age 5 years, and ii) understanding the required differences in analytical approach to assess the proportion meeting the physical activity guidelines. Figure 1 helps overcome the first challenge as it illustrates the impact of the progression towards the MVPA requirement on the results: 84% meet the guideline but only 11% accumulate 60 minutes of MVPA within those 3 hours. To remain consistent with the age brackets of the physical activity guidelines (i.e., 0–4 years and 5–17 years) as well as how children aged 6 years and older have been assessed previously in the CHMS [21], we used a probability function to estimate the proportion of 5 year olds meeting the guideline. This analytical approach is different to simply looking at meeting the target on all valid days, which is how 3–4 year olds were assessed. If we had assessed 5 year olds in this way, the data would have indicated that 7% (instead of 14%) met the physical activity guideline. The probability function is more robust when assessing low levels of adherence [21,23] and that is why we presented the 14% value as the primary finding for 5 year olds in this analysis.

## Conclusions

The majority of 3 and 4 year old children in Canada are meeting current physical activity guidelines; however, only 18% are meeting their age-specific screen time recommendation within the sedentary behavior guideline. The opposite trend was observed in 5 year old children with 14% meeting their age specific physical activity guideline and the majority (81%) meeting their screen time recommendation within sedentary behavior guideline. Overall, very few Canadian children are meeting both guidelines. Promoting physical activity while reducing sedentary behavior is important at all stages of life. The findings of the present study indicate that there remains significant room for improvement in these behaviors among young Canadian children.

## Competing interests

The authors declare that they have no competing interest.

## Authors’ contributions

RCC conceived the idea for the manuscript, completed background analyses to inform the analysis plan, led the research team and drafted the manuscript. DG oversaw the project and completed all data reduction and statistical analysis procedures. IJ and VC provided expert analytical advice relating to accelerometry and epidemiology and provided considerable insight into the interpretation of the results. KBA, BWT and MST were involved in the analytical planning and provided critical insight during the drafting and review of the manuscript. All authors were involved in the planning of the manuscript, including conception and analytical plan. All authors have reviewed the manuscript and approved the final submitted version.
